# The Global Burden of Childhood Coeliac Disease: A Neglected Component of Diarrhoeal Mortality?

**DOI:** 10.1371/journal.pone.0022774

**Published:** 2011-07-26

**Authors:** Peter Byass, Kathleen Kahn, Anneli Ivarsson

**Affiliations:** 1 Umeå Centre for Global Health Research, Department of Public Health and Clinical Medicine, Umeå University, Umeå, Sweden; 2 MRC/Wits Rural Public Health and Health Transitions Research Unit (Agincourt), School of Public Health, Faculty of Health Sciences, University of the Witwatersrand, Johannesburg, South Africa; Ludwig Maximilian University of Munich, Germany

## Abstract

**Objectives:**

Coeliac disease has emerged as an increasingly recognised public health problem over the last half-century, and is now coming to be seen as a global phenomenon, despite a profound lack of globally representative epidemiological data. Since children with coeliac disease commonly present with chronic diarrhoea and malnutrition, diagnosis is often overlooked, particularly in poorer settings where children often fail to thrive and water-borne infectious diarrhoeas are common. This is the first attempt to make global estimates of the burden of coeliac disease in childhood.

**Methods:**

We built a relatively crude model of childhood coeliac disease, incorporating estimates of population prevalence, probability of non-diagnosis, and likelihood of mortality among the undiagnosed across all countries from 1970 to 2010, based around the few available data. All our assumptions are stated in the paper and the model is available as a supplementary file.

**Findings:**

Our model suggests that in 2010 there were around 2.2 million children under 5 years of age living with coeliac disease. Among these children there could be 42,000 deaths related to coeliac disease annually. In 2008, deaths related to coeliac disease probably accounted for approximately 4% of all childhood diarrhoeal mortality.

**Conclusions:**

Although coeliac disease may only account for a small proportion of diarrhoeal mortality, these deaths are not preventable by applying normal diarrhoea treatment guidelines, which may even involve gluten-based food supplements. As other causes of diarrhoeal mortality decline, coeliac disease will become a proportionately increasing problem unless consideration is given to trying gluten-free diets for children with chronic diarrhoea and malnutrition.

## Introduction

Over the last half-century, research has increasingly revealed the nature and extent of coeliac disease, particularly among European populations, but there remains much to be learnt about this disease on a global scale [1]–[3]. Coeliac disease is a systemic autoimmune syndrome involving a gluten-induced chronic inflammation of the small bowel mucosa, with extensive short- and long term negative health consequences if untreated [4]. Symptomatology can vary for an individual over time, and often mimics other diseases, which, combined with low global awareness of the disease, results in many cases remaining undiagnosed or being ineffectively treated. Examples of signs and symptoms are malabsorption with diarrhoea and consequent under-nutrition, short stature, anaemia, stomach pain, and increased incidence of many infectious diseases. The disease is effectively treated by life-long exclusion of all foods containing any amount of gluten-bearing cereals (wheat, rye or barley), while all other foods, including principal components of staple diets in many societies such as rice and maize, are completely safe. Adopting a gluten-free diet, either following clinical diagnosis or in response to symptoms, resolves coeliac disease enteropathy, and thereby eliminates associated health risks. Thus, from a public health perspective, the at-risk group comprises those living with coeliac disease that is not diagnosed as such.

The sporadic data that do exist from Africa, Asia and Latin America indicate that coeliac disease is by no means confined to European populations [3], [5]–[15]. In contrast, we were unable to find any reports of populations free from coeliac disease. A recent study of worldwide in-migrants to Sweden also showed that non-European ethnicities were susceptible to coeliac disease but at rates ranging from 41% to 125% of that of the native population [16]. As part of globalisation, regular consumption of foods containing wheat is becoming increasingly common, even in traditionally maize or rice-consuming societies, possibly leading to an increased burden of coeliac disease. Wheat is also commonly supplied by international food aid programmes. Wheat consumption data for 2000 showed that the Middle East and Western Europe had the highest levels (147 kg/person/year and 102 kg/person/year respectively), while other regions were closer to 60 kg/person/year [17]. Even in Europe, where coeliac disease was first recognised in the mid-20^th^ century, data from community-based screening studies suggest that rates of routine clinical diagnosis remain far from complete [3], [9], [18], [19] though they are improving over time [20]. In settings where diarrhoeal infections remain as common causes of childhood morbidity and mortality, it is not difficult to postulate that coeliac disease is a cause that can easily escape attention [5]. At the same time it is reasonable to suppose that continuing non-diagnosis may well lead to severe illness and death in affected children, despite symptomatic treatments such as oral rehydration fluids, nutritional supplements (possibly containing gluten) and antibiotics [21]. Inevitably there are very few data on the mortality risk of undiagnosed coeliac disease, but one historical study from the USA estimates a four-fold risk [22]. Historic data (from before the discovery of gluten-free diet for managing coeliac disease) from the United Kingdom [23] suggested a childhood case-fatality rate of up to 30% in the 1930s, when mortality between the ages of 1 and 5 years was around 3% (www.mortality.org), suggesting a mortality risk of up to 10.

This combination of factors around coeliac disease and its under-diagnosis has led us to consider how much it might contribute to the global burden of childhood morbidity and mortality. Since the global data are very sparse, at this stage the only option was to model estimates of what numbers may be involved and in which regions of the world the consequences may be greatest. Making global estimates of this kind is a somewhat fraught process, and findings need to be handled with care [24]. Inevitably this process has involved making a number of assumptions which could well be contested, but our aim in this paper is to set out our reasoning and findings in a transparent fashion, accompanied by a supplementary file containing our detailed data and results, so that at least the possible global burden of childhood coeliac disease can be further debated and investigated.

## Methods

**Figure 1:**
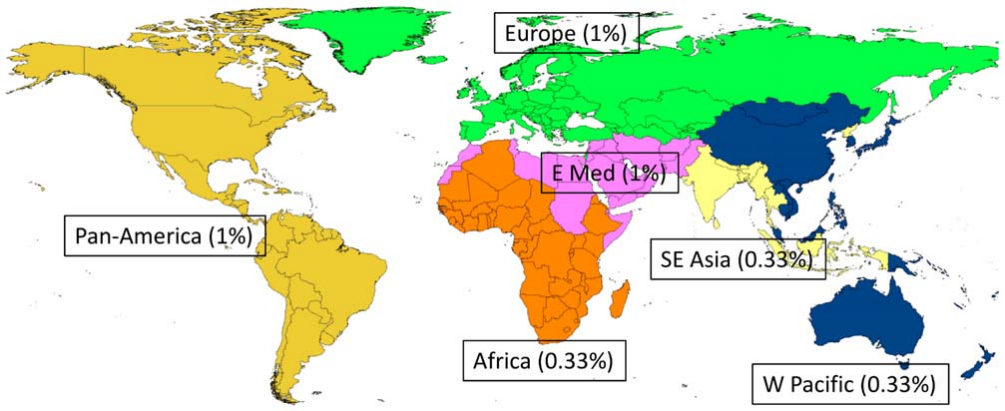
World map by WHO Regions, as used as the basis for modelled estimates, showing underlying assumptions about the population prevalence of childhood coeliac disease.

As a starting point we have taken established national estimates of under-5 population and mortality for the entire world from 1970 to 2010 [25], [26], remembering that such estimates are themselves under frequent revision and update [27]. In addition recent estimates of child mortality for 2008 give specific country data for diarrhoeal deaths [28]. Different sources of various estimates use a multiplicity of regional groupings of countries, and we have chosen to follow the WHO regional groupings for the sake of widespread comparability, even though this contains some geographical inconsistencies such as, for example, the political necessity to have the Republic of Korea and adjoining Democratic People's Republic of Korea in different regions. The WHO regions are illustrated in Figure 1.

Since data on population prevalences of coeliac disease are very limited or non-existent for many countries, we had to make relatively crude assumptions about regional prevalence rates, as also shown in Figure 1, based on known point-prevalence estimates, taking 1% except for Africa, South-east Asia and the Western Pacific, which were taken as 0.33% [3], [5]–[16]. The 1% value is within the typical range of population-based measurements, where these have been made; the 0.33% value for Africa and South-East Asia was chosen as a more conservative estimate given the general lack of data from those areas but some evidence of lower disease prevalence than elsewhere.

The second area in which major assumptions were needed was the extent of non-diagnosis of coeliac disease at different times and places, conceptualised in equation (I) as the probability of an individual with coeliac disease being undiagnosed in a particular time and context:
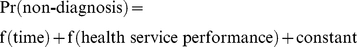
(I)


In 1970, at the start of the period covered by the data used here, awareness of coeliac disease was not widespread and throughout the world probabilities of non-diagnosis were high [19], [29]. This has gradually changed over time, but it is still reasonable to assume that settings with relatively basic health services have only progressed slowly with respect to rates of coeliac disease diagnosis. Although WHO has worked with an index of health service performance, this is not available with variations by country and time [30]. Since there is no absolute global measure of health service performance over time, we have used national cumulative under-5 mortality proportions by year and country as a proxy. Hence we have developed the general statement of equation (I) into more specific terms in equation (II):
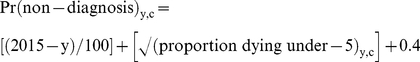
(II)which was calculated separately for each year y and country c, with a capped value of 1.

It then follows (equation III) that the number of children with undiagnosed coeliac disease can be estimated for each year y and country c as:
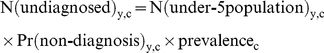
(III)


The third major assumption concerns the relative risk of mortality for children with undiagnosed coeliac disease compared to children in general in the same country and year. There are by definition hardly any data available for this, but we considered it reasonable to assume that it would be related to the annual probability of all-cause deaths for children between 1 and 4 years of age (annual _5_q_1_) for a particular year y and country c, and we have taken a relative risk (RR) of 5, which is also close to the one published estimate of mortality risk [22]. By excluding infancy, this assumption avoids the potentially distorting effect of variations in neonatal and infant mortality. Thus the annual numbers (N) of childhood deaths attributable to undiagnosed coeliac disease was estimated for each year y and country c according to equation (IV):
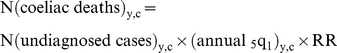
(IV)


It then follows that the case-fatality rate (CFR) for undiagnosed coeliac disease can be estimated from equation (V) for each year y and country c as:
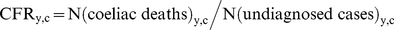
(V)and the cause-specific mortality rate (CSMR) for coeliac disease per 1,000 children at the population level can be estimated from equation (VI) as:
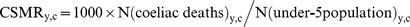
(VI)


Data were managed using Microsoft FoxPro v9 and analysed using Stata v10, and the detailed data by year and country are presented in spreadsheet format as supplementary material (Dataset S1).

## Results

**Figure 2:**
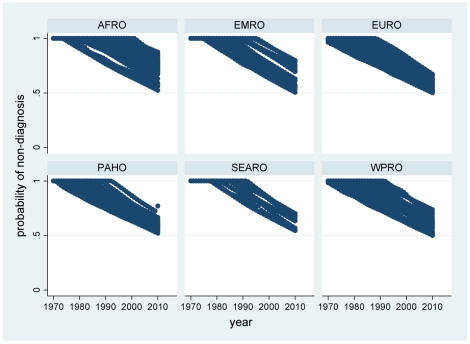
Scatter plots of probabilities of non-diagnosis of coeliac disease by country and year, grouped by WHO region. (AFRO African region, EMRO Eastern Mediterranean region, EURO European region, PAHO Pan-American region, SEARO South-east Asian region, WPRO Western Pacific region; as shown in Figure 1).

**Figure 3:**
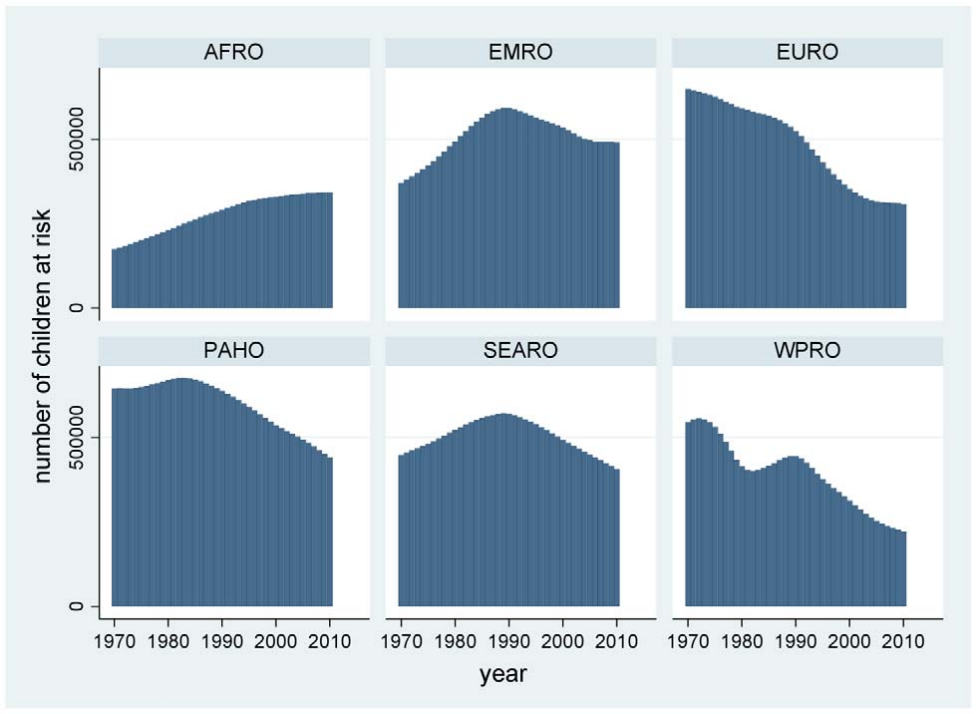
Estimated numbers of under-5 children with undiagnosed coeliac disease, by WHO region and year. (AFRO African region, EMRO Eastern Mediterranean region, EURO European region, PAHO Pan-American region, SEARO South-east Asian region, WPRO Western Pacific region; as shown in Figure 1).

**Figure 4:**
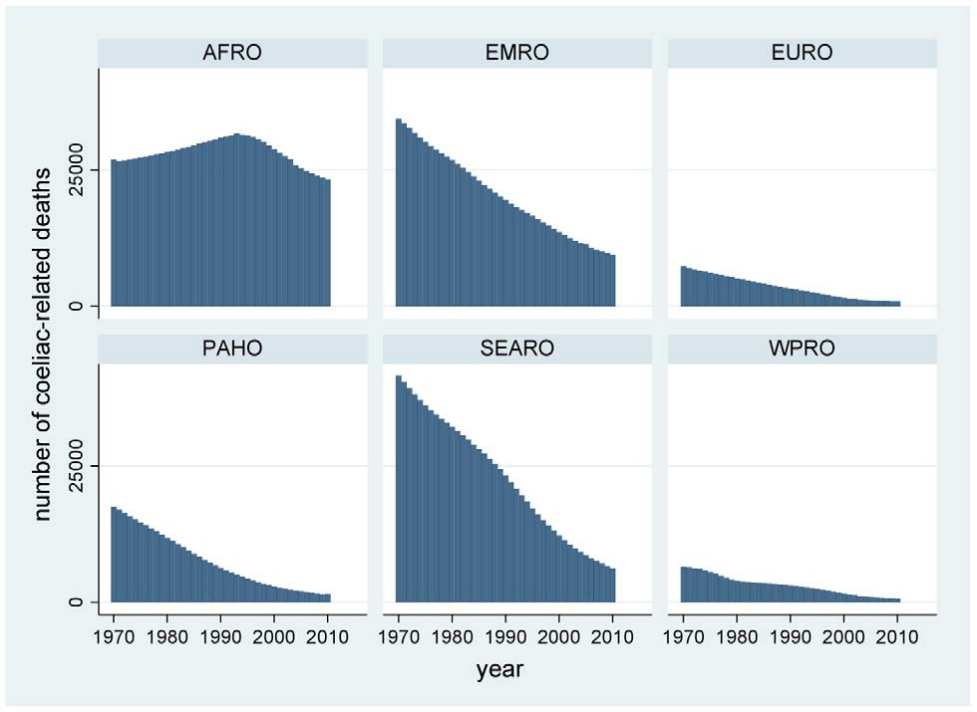
Estimated numbers of coeliac disease related under-5 deaths by WHO region and year. (AFRO African region, EMRO Eastern Mediterranean region, EURO European region, PAHO Pan-American region, SEARO South-east Asian region, WPRO Western Pacific region; as shown in Figure 1).

**Figure 5:**
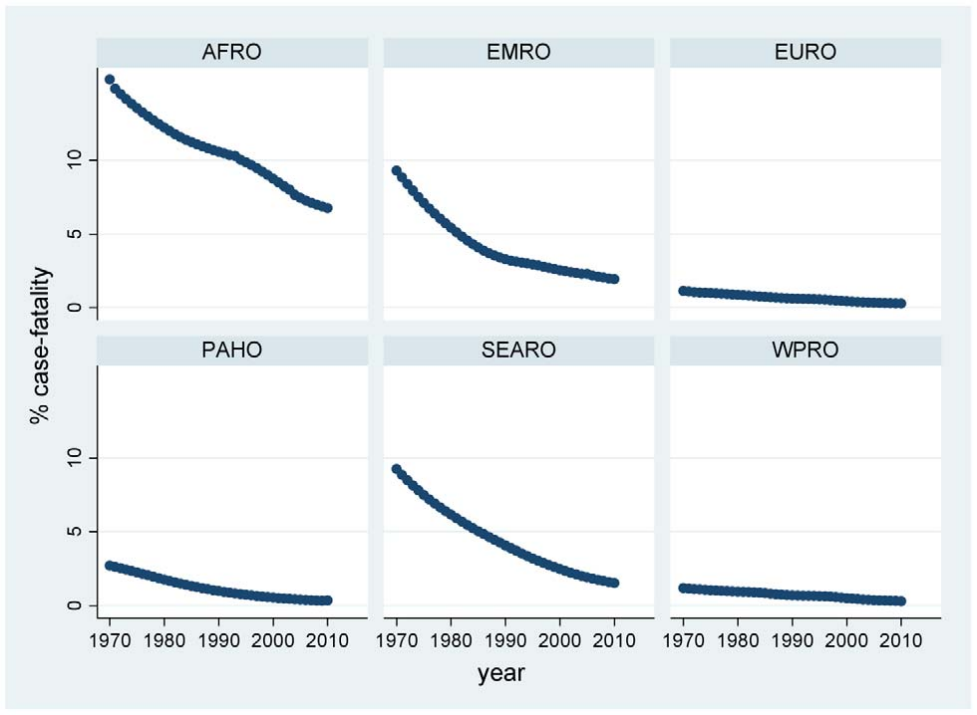
Estimates of coeliac disease case-fatality rates among under-5 children by WHO region and year. (AFRO African region, EMRO Eastern Mediterranean region, EURO European region, PAHO Pan-American region, SEARO South-east Asian region, WPRO Western Pacific region; as shown in Figure 1).

**Figure 6:**
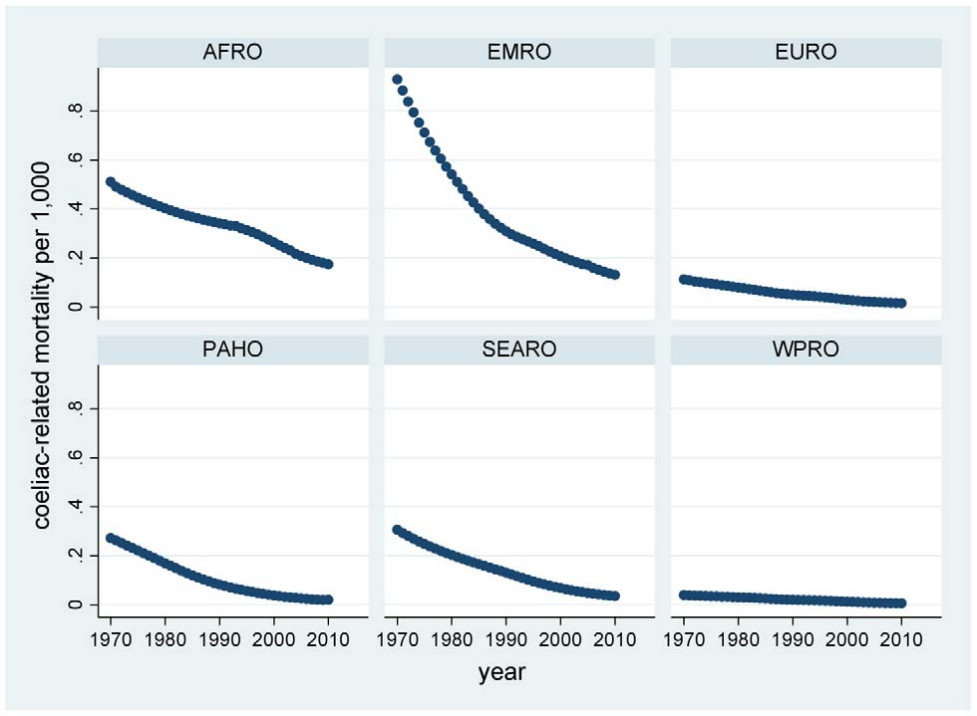
Estimates of coeliac disease related mortality rates in under-5 populations by WHO region and year. (AFRO African region, EMRO Eastern Mediterranean region, EURO European region, PAHO Pan-American region, SEARO South-east Asian region, WPRO Western Pacific region; as shown in Figure 1).

Results of the modelling by year and country are presented here according to WHO regions. The complete by year and country data are available in the supplementary file (Dataset S1) for readers who may wish to classify countries in other ways. The distributions of probabilities of non-diagnosis of childhood coeliac disease over time and country, calculated according to equation (II), are presented by region in Figure 2. These were then used to calculate corresponding numbers of undiagnosed children according to equation (III), which are shown by region in Figure 3. Figure 4 shows the estimated number of under-5 deaths by region attributable to coeliac disease, calculated according to equation (IV). Global totals for 2010 amount to 2.2 million undiagnosed children, of whom 42,000 may die annually (1.9%). Figures 5 and 6 show regional estimates over time for case fatality rates among children with undiagnosed coeliac disease and cause-specific mortality rates for coeliac disease at the population level, respectively, calculated according to equations (V) and (VI).

**Figure 7:**
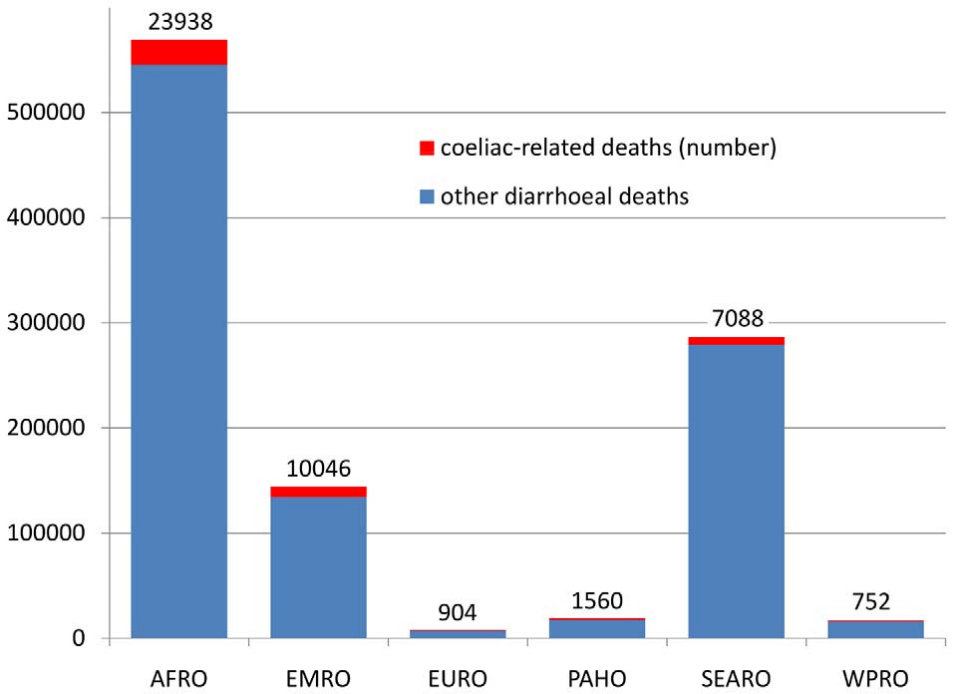
Estimated under-5 diarrhoeal deaths in 2008 by WHO region, showing the estimated coeliac-related component (red bars and numbers of cases). (AFRO African region, EMRO Eastern Mediterranean region, EURO European region, PAHO Pan-American region, SEARO South-east Asian region, WPRO Western Pacific region; as shown in Figure 1).

Estimates of the proportions of under-5 deaths attributable to diarrhoea by country are available for 2008 from the CHERG group [28]. These were used alongside our modelled results for 2008 to estimate, by region, the proportion of under-5 diarrhoeal mortality that could have been coeliac-related during that year. These results are shown in Figure 7. Globally these figures amounted to 44,300 coeliac-related deaths out of 1.04 million diarrhoea-related deaths (4.2%).

As identified above, there are three important areas in the model where key assumptions had to be made: the population prevalence of coeliac disease; the probability of non-diagnosis of coeliac disease; and the relative risk of mortality associated with undiagnosed coeliac disease, as well as uncertainties in the global child mortality estimates used in our equations. We have undertaken uncertainty and sensitivity analyses to reflect variations in modelled outcomes accordingly. The global child mortality estimates by year and country which are used in the model as described above have well-established uncertainty bounds [28]. Reworking the model using low bounds and high bounds respectively results in an uncertainty range from 36,000 to 49,000 around our estimate of 42,000 deaths in 2010 (approximately ±15%). Since there is no reasonable evidence base for constructing uncertainty bounds around our assumptions about prevalence, non-diagnosis and case fatality, we have use a sensitivity analysis approach to illustrate the effects of variations in these parameters. Table 1 shows the results of sensitivity analyses around these assumptions, based on overall figures of undiagnosed children and deaths related to coeliac disease for 1990, 2000 and 2010.

**Table 1:** Sensitivity analyses for key estimates of coeliac-related morbidity and mortality, applied to numbers of undiagnosed cases and deaths in 1990, 2000 and 2010.

Model parameter	Model outputs (in 000s)					
	1990		2000		2010	
	cases	deaths	cases	deaths	cases	deaths
**Model as presented**	**3,062**	**86.1**	**2,560**	**60.5**	**2,211**	**41.9**
1% prevalence globally	5,715	202.1	4,866	1,469	4,182	102.9
0.5% prevalence globally	2,857	101.1	2,433	734.5	2,091	51.5
Pr(non-diagnosis) ↑ 50%	3,450	88.8	3,332	68.7	3,460	55.1
Pr(non-diagnosis) ↓ 50%	1,543	44.5	1,280	30.3	1,105	20.9
mortality relative risk ↑ 50%	3,062	172.2	2,560	121.1	2,211	83.8
mortality relative risk ↓ 50%	3,062	43.1	2,560	30.3	2,211	21.0

## Discussion

Our results suggest that coeliac disease is a neglected global public health problem, contributing to avoidable morbidity and mortality among young children. Based on the relatively conservative estimates made in our model, around 2.2 million children under five years of age today probably have undiagnosed coeliac disease. Around 42,000 childhood deaths annually could still be due to coeliac disease, despite in some of those children diarrhoeal symptoms being treated according to WHO recommendations, which as yet do not take into account the possibility of some children needing a gluten-free diet to alleviate diarrhoea [21].

Considering these results, and despite the scarcity of underlying global data, it appears that coeliac disease represents an appreciable global problem. We have had to make quite sweeping assumptions to build the model, but as far as possible these are based on such evidence as is actually available and are relatively conservative compared with, for example, some high point-prevalence measures [8], [31]. The sensitivity analyses (presented in Table 1) demonstrate that variations in our key assumptions have approximately linear effects on output parameters, indicating a rather stable model, and carrying the uncertainty bounds associated with the global child mortality estimates through the model had a relatively modest effect on the overall outcome. An initial version of our model, built from the previous generation of global mortality estimates, also gave rather similar overall findings (data not shown), which constitutes another kind of indirect sensitivity analysis.

Some of the assumptions that we have made in modelling these estimates can certainly be challenged; one of our intentions in publishing this model is precisely to stimulate such debate. Very few data are available on the population-based prevalence of coeliac disease in settings where childhood morbidity and mortality arising from diarrhoea and malnutrition are common. Our assumptions on global prevalence are well within the range of known point-prevalence estimates. Regional variations in genetic profiles and environmental and lifestyle factors, such as the age when gluten-containing foods are introduced to infants and possibly the amounts involved, probably contribute to variations in coeliac disease prevalence [18]. However, at the moment this knowledge is insufficiently complete and established to be useful in global modelling. Non-diagnosis of children with classic symptoms of coeliac disease is subjectively much more likely in settings where diarrhoeal disease and malnutrition is common, which we believe may be reasonably well captured by relating it to under-5 mortality as a proxy, although this may not capture local details such as referral hospitals which may achieve higher rates of diagnosis. Then it seems reasonable to assume that the risk of mortality associated with undiagnosed coeliac disease is directly related to childhood population mortality risks in the same settings–the question being the extent of the relative risk. Clearly there are no contemporary or future studies concerning the relative mortality risk of non-diagnosis, though the limited historical evidence suggests a relative risk of 5 is not unreasonable [22], [23] More empirical data from a wide geographic range are clearly needed to confirm or revise the global basis of many of the assumptions in our model; this is simply a starting point.

Closer examination of the model outputs suggests distinctly different patterns and dynamics of coeliac disease in different regions. Africa is the only region with a substantial continuing growth in under-5 population, and these demographics mean that any childhood disease in Africa may be subject to increase in absolute terms unless countermeasures also become increasingly effective (Figure 3). Since this growth is coupled with some of the world's least effective health services, the burden of childhood mortality is likely to be high. It is not surprising therefore to see that the present-day coeliac-related case fatality rate is by far the highest in Africa (Figure 5).

Established guidelines in Europe for diagnosing coeliac disease involves firstly analyses of serological markers (antihuman tissue transglutaminase of isotype IgA), and, if these are elevated, a secondary evaluation of a small bowel mucosal specimen to reveal enteropathy, followed by normalisation of serological markers and symptoms consequent upon a strict gluten-free diet. Although coeliac disease has been mostly documented in industrialised countries, it is important to note that our model suggests that its biggest burden of morbidity and mortality falls in Africa and Asia. Given the relatively basic nature of diagnostic facilities in these settings, it is likely that relatively few cases will be definitively diagnosed according to the above guidelines, and it may be appropriate to develop public health strategies involving at least temporary exclusion of dietary gluten (i.e. all wheat-based foods) as presumptive diagnosis and management for children who present with persistent diarrhoea, malnutrition or failure to thrive. In most of Africa and Asia maize- and rice-based foods are relatively easily available alongside wheat-based products, so that the provision of special foods such as gluten-free bread and pasta are probably not necessary. However, care needs to be taken in settings with high HIV-prevalence and poor prevention of mother-to-child transmission (PMTCT) programmes since diarrhoea, malnutrition and failure to thrive is a common presenting clinical picture in paediatric HIV/AIDS as well as in coeliac disease. There is no population-based literature on interactions between HIV and coeliac disease. Thus likely HIV infection should be excluded before trying a gluten-free diet in such children so as not to further compromise nutritional status or confuse nutritional messages given to caregivers of HIV positive children.

It is curious to note that a recent major review of research priorities on childhood diarrhoea [32] does not even mention coeliac disease as a potential distinct cause of childhood diarrhoeal deaths. Work on childhood diarrhoea from the Child Health Epidemiology Reference Group (CHERG) also does not appear to consider coeliac disease as a distinct aetiology for diarrhoea [33], even though appreciable proportions of diarrhoeal disease are not associated with specific aetiologies. If advances in preventing other causes of diarrhoeal death were to be increasingly successful, then one would have to assume that the proportion of coeliac-related mortality out of all diarrhoeal causes would rise. It could thus represent an increasingly large and intractable proportion of global childhood diarrhoeal mortality in the future, in the absence of increased awareness and better patient management strategies. Recognising this also has implications for the distribution of wheat as emergency food aid, particularly to malnourished children. It is therefore very important in terms of public health strategy to ensure that coeliac disease is put firmly onto the paediatric diagnostic agenda on a global basis.

## Supporting Information

Dataset S1 **Global coeliac disease model, by country and year.**
(XLS)
